# Metabolite Signature in the Carriers of Pathogenic Genetic Variants for Cardiomyopathy: A Population-Based METSIM Study

**DOI:** 10.3390/metabo12050437

**Published:** 2022-05-13

**Authors:** Rowmika Ravi, Lilian Fernandes Silva, Jagadish Vangipurapu, Maleeha Maria, Joose Raivo, Seppo Helisalmi, Markku Laakso

**Affiliations:** 1 Institute of Clinical Medicine, Internal Medicine, University of Eastern Finland, 70210 Kuopio, Finland; rowmika.ravi@uef.fi (R.R.); lilianf@uef.fi (L.F.S.); jagadish.vangipurapu@uef.fi (J.V.); joose.raivo@uef.fi (J.R.); seppo.helisalmi@uef.fi (S.H.); 2 A.I. Virtanen Institute for Molecular Sciences, University of Eastern Finland, 70210 Kuopio, Finland; maleeha.maria@uef.fi; 3 Department of Medicine, Kuopio University Hospital, 70210 Kuopio, Finland

**Keywords:** hypertrophic cardiomyopathy, dilated cardiomyopathy, metabolites, metabolomics

## Abstract

Hypertrophic (HCM) and dilated (DCM) cardiomyopathies are among the leading causes of sudden cardiac death. We identified 38 pathogenic or likely pathogenic variant carriers for HCM in three sarcomere genes (*MYH7*, *MYBPC3*, *TPMI*) among 9.928 participants of the METSIM Study having whole exome sequencing data available. Eight of them had a clinical diagnosis of HCM. We also identified 20 pathogenic or likely pathogenic variant carriers for DCM in the *TTN* gene, and six of them had a clinical diagnosis of DCM. The aim of our study was to investigate the metabolite signature in the carriers of the pathogenic or likely pathogenic genetic variants for HCM and DCM, compared to age- and body-mass-index-matched controls. Our novel findings were that the carriers of pathogenic or likely pathogenic variants for HCM had significantly increased concentrations of bradykinin (des-arg 9), vanillactate, and dimethylglycine and decreased concentrations of polysaturated fatty acids (PUFAs) and lysophosphatidylcholines compared with the controls without HCM. Additionally, our novel findings were that the carriers of pathogenic or likely pathogenic variants for DCM had significantly decreased concentrations of 1,5-anhydrogluticol, histidine betaine, N-acetyltryptophan, and methylsuccinate and increased concentrations of trans-4-hydroxyproline compared to the controls without DCM. Our population-based study shows that the metabolite signature of the genetic variants for HCM and DCM includes several novel metabolic pathways not previously described.

## 1. Introduction

Cardiomyopathies are among the leading causes of sudden cardiac death. The prevalence of hypertrophic cardiomyopathy (HCM) has been estimated to be 1 in 500 and dilated cardiomyopathy (DCM) 1 in 2500 individuals worldwide [1,2,3]. The definition of HCM is based on an increase in the left ventricular (LV) wall thickness ≥15 mm in the absence of other causes of LV hypertrophy such as hypertension, valvular diseases, or coronary artery disease [3]. DCM is defined as a left ventricular dilatation that leads to systolic dysfunction in the absence of any other abnormal loading conditions or coronary artery disease [3].

HCM and DCM have autosomal dominant inheritance with strong phenotypic heterogeneity and incomplete penetrance [4,5]. Mutations in the genes encoding sarcomeric proteins (*ACTC1*, *MYBPC3*, *MYH7*, *MYL2*, *MYL3*, *TNNI3*, *TNNT2*, *TPM1*) are the most important genes for HCM. Very rarely (<2%) mutations in non-sarcomeric genes cause HCM [6]. We previously found pathogenic or likely pathogenic mutations in 38% of 382 clinically diagnosed patients with HCM [7]. Other studies have found causal variants in 30–60% of the patients with HCM [8]. Finnish three founder mutations, Gln1061Ter of *MYBPC3*, Arg1053Gln of *MYH7* and Asp175Asn of *TPM1*, and a prevalent mutation Val606Met of *MYH7* account for 28% of HCM cases in Finns [7]. Genes that are causing DCM encode components of sarcomere (*TTN*, *MYH7*, *TNNT2*, *TNNI3*, *TPM1*, *ACTC1*), sarcomere associated proteins (*PLN*, *BAG3*), nuclear membrane (*LMNA*, *EMD*), cytoskeleton (*DES*), outer cellular membrane, extracellular matrix *(DMD)*, ion channels (*SCN5A*), mitochondrial proteins (*TAZ*, *DNAJC19*), and splice-regulating proteins (*RBM20*) [8]. Pathogenic or likely pathogenic variants explain about 30% of the cases of DCM. Importantly, HCM and DCM are not defined by specific genetic mutations, but by specific morphological and functional alterations in the heart [9,10].

Myocardial energy metabolism is changed in cardiomyopathies. HCM is caused by altered biophysical properties of cardiomyocytes, disturbed calcium handling and abnormal cellular metabolism [11,12]. The primary defect is a sarcomere mutation, but clinical phenotypes are determined by genetic, epigenetic, and environmental factors [9]. A hallmark of pathological cardiac hypertrophy in patients with HCM is the reversion to fetal gene expression associated with reductions in fatty acid oxidation and an increase in glucose utilization by an enhancement of glucose uptake and glycolysis [12].

Metabolomics allows a detailed characterization of metabolic phenotypes and enables *precision medicine* approach including the characterization of metabolic derangements that underlie disease, discovery of new therapeutic targets and biomarkers that may be used to diagnose a disease or monitor the course of therapy [9,13,14]. Application of metabolomics in studies of cardiomyopathies is still limited although the current methods based on mass spectrometry allow the screening over 1000 plasma metabolites. An advantage of studying plasma metabolites as biomarkers for HCM and DCM is that cardiomyopathies have a well-established genetic basis, and that cardiovascular risk factors do not play a major role in the risk of these diseases. Our large Finnish population-based cohort the Metabolic Syndrome In Men (METSIM) Study [15] including 10,197 men is ideal to investigate metabolite signature in carriers of pathogenic variants for cardiomyopathies.

## 2. Results

### 2.1. Identification of Genetic Variants for HCM and DCM

We identified the pathogenic or likely pathogenic variants for HCM in our exome sequencing data in the following eight genes associated with HCM based on previous studies: *ACTC1*, *MYBPC3*, *MYH7*, *MYL2*, *MYL3*, *TNNI3*, *TNNT2*, and *TPM1* [16]. We found 38 pathogenic or likely pathogenic genetic variants for HCM in three sarcomere genes. For each carrier of pathogenic or likely pathogenic genetic variants for HCM, we selected five age- and BMI-matched controls.

There were no statistically significant differences in clinical and laboratory measurements between cases and controls (Table 1). The prevalence of the pathogenic variants for HCM was 0.37% of the participants, suggesting that 1 of 270 participants was a carrier of a pathogenic variant for HCM in our population (Table 2). The most frequent pathogenic genetic variant was Arg1053Gln of *MYH7*, found in 24 participants. The frequencies of other pathogenic variant carriers were low: Arg941His of *MYH7* (*n* = 4), Gln1061Ter of *MYBPC3* (*n* = 3), c.655-2A > C of *MYBPC3* (*n* = 4), Gly853fs of *MYBPC3* (*n* = 1), and Asp175Asn of *TPM1* (*n* = 2).

Eight of thirty-six participants had a clinical diagnosis of HCM based on Kuopio University Hospital medical records. We compared the metabolite signature between participants having a clinical diagnosis of HCM (*n* = 8) with participants who did not have a clinical diagnosis of HCM (*n* = 28). We did not find statistically significant differences in metabolite concentrations between these groups.

We screened the pathogenic or likely pathogenic variants for DCM in our exome sequencing data in the following sixteen genes associated with DCM in previous studies: *ACTC1*, *BAG3*, *DES*, *DMD*, *DNAJC19*, *EMD*, *LMNA*, *MYH7*, *PLN*, *RBM20*, *SCN5A*, *TAZ*, *TNNI3*, *TNNT2*, *TPM1*, and *TTN* [8,17]. We included in our analyses only the variants located in the protein coding regions or in canonical splice sites. We found 20 pathogenic or likely pathogenic genetic variants only in the *TTN* gene (Table 2). These variants caused terminal codon in fifteen cases; one was a splice variant, and four were pathogenic duplications. For each carrier of the pathogenic or likely pathogenic genetic variants for DCM, we selected five age- and BMI-matched controls. There were no statistically significant differences in clinical and laboratory measurements between the cases and controls (Table 1). The prevalence of pathogenic variants for DCM was 0.20%, suggesting that 1 of 500 participants was a carrier of a pathogenic variant.

### 2.2. Identification of Metabolites Associated with HCM Pathogenic Variants

We found 23 novel metabolites associated with the HCM pathogenic variants. Compared to the age- and BMI-matched controls, the carriers of these variants had increased concentrations of metabolites in the amino acid pathway, including dimethylglycine, vanillactate, and des-Arg 9-bradykinin. We also found that the carriers of the HCM variants had decreased concentrations of 17 lipids, especially fatty acids and lysophosphatidylcholines, compared to controls (Table 3).

### 2.3. Identification of Metabolites Associated with DCM Pathogenic Variants

We found that the carriers of the DCM pathogenic variants had six novel associations with metabolites belonging to the carbohydrate, lipid, and amino acid pathways. We found decreased concentrations of 1,5-anhydrogluticol (1,5-AG, glycolysis pathway), bilirubin (lipid pathway), histidine betaine, N-acetyltryptophan, and methylsuccinate (amino acid pathway) in the carriers of the DCM pathogenic variants compared to the correspondent controls. We also found increased concentrations of trans-4-hydroxyproline and confirmed a previously reported association with homoarginine in the carriers of DCM pathogenic variants compared to the controls (Table 4).

Six of twenty participants had a clinical diagnosis of DCM based on Kuopio University Hospital medical records. We compared the metabolite signature between participants with a clinical diagnosis of DCM (*n* = 8) with participants without a clinical diagnosis of DCM (*n* = 28) but did not find statistically significant differences in metabolite concentrations between these groups.

## 3. Discussion

We identified 38 pathogenic or likely pathogenic genetic variants for HCM in three sarcomere genes (*MYH7 MYBPC3*, *TPM1*) among 9928 participants in a population-based METSIM study. Only eight of them had clinically diagnosed HCM based on Kuopio University Hospital medical records. We found 20 pathogenic or likely pathogenic genetic variants in the *TTN* gene for DCM, and almost all of them were stop codon or frameshift variants, in agreement with previously published studies [18,19]. Six of twenty participants had a clinical diagnosis of DCM. 

Our study was based only on the carriers of pathogenic or likely pathogenic genetic variants for HCM and DCM, and therefore, it is likely that these individuals had clinically mild cases of cardiomyopathies. This makes our study population more homogenous than studies based on the clinical diagnosis of cardiomyopathies and gives an excellent opportunity to investigate the effects of HCM and DCM variants on the metabolic signature of these diseases.

Previous metabolomics studies in HCM have been heterogenous, comparing the metabolite signature between obstructive and nonobstructive HCM [20,21], or focusing on myocardial biopsies [12] or the metabolomics profile of the carriers of a single mutation [22]. Our study focuses on the carriers of pathogenic variants causing HCM who were carefully matched for the controls with respect to age and BMI. Our control groups were five times larger than cardiomyopathy groups, increasing the power of the statistical analyses.

**Figure 1:**
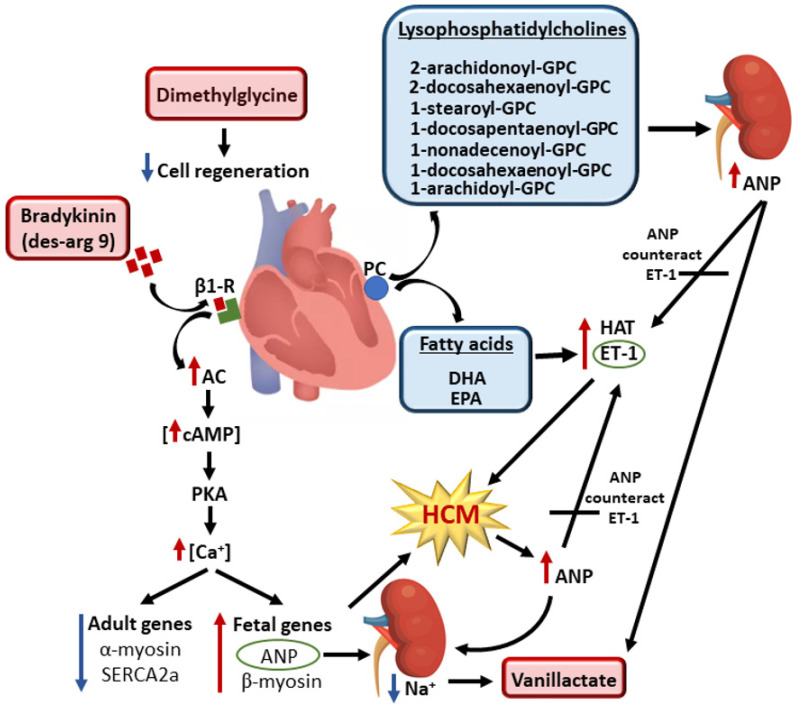
Metabolites involved in the cell signaling that regulates hypertrophy of cardiomyocytes in the carriers of pathogenic or likely pathogenic genetic variants for HCM. Red boxes indicate increased concentrations and blue boxes decreased concentrations of the metabolites in the carriers of genetic variants compared to the controls. Bradykinin activates β1-receptors, resulting in high intracellular concentrations of calcium and suppression of adult and activation of fetal gene expression. This leads to enhanced cardiomyocyte contractility, impaired relaxation, induced hypertrophic remodeling, and a release of ANP as a response to hemodynamic loading. Increased ANP concentrations lead to an increase in sodium excretion, which increases vanillactate concentrations. Hydrolysis of PC generates lysophosphatidylcholines (LPC) and fatty acids. A decrease in lysophosphatidylcholine concentration increases ANP concentrations. Low concentrations of EPA and DHA activate HAT and ET-1, triggering hypertrophy of cardiomyocytes. Released ANP antagonizes ET-1 and restores hemodynamic stability. Dimethylglycine impairs cell regeneration and contributes to the development of HCM. Abbreviations: AC, adenyl cyclase; ANP, atrial natriuretic peptide; β1-R, β1 receptor; Ca, calcium; cAMP, cyclic adenosine monophosphate; DHA, docosahexaenoate; EPA, eicosapentaenoate; ET-1, endothelin 1; GPC, glycerophospatidylcholine; HAT, histone acyltransferase; Na, sodium; LPC, lysophosphatidylcholine; PC, phosphatidylcholine; PKA, protein kinase A; SERCA2a, Ca^2+^-ATPase 2a.

We found statistically significant differences between the HCM variant carriers and controls in several metabolites belonging to the amino acid, lipid, and carbohydrate classes (Figure 1). Our novel findings were that we found increased concentrations of des-arg(9)-bradykinin, vanillactate, and dimethylglycine in participants with the pathogenic HCM variants compared to the controls. Des-arg(9)-bradykinin is an active metabolite of bradykinin and stimulates β1 receptors [23]. The β-adrenergic signaling pathway is one of the pathways that mediates cardiac hypertrophy and plays a central role in the pathophysiology of heart failure [24,25]. Chronic stimulation of β1 receptors induces cardiac hypertrophy in in vitro experiments and in animal studies by activating adenylyl cyclase via Gs proteins [26]. Consequently, the cellular concentration of cyclic AMP (cAMP) increases and stimulates protein kinase A, which modulates the calcium channels [27]. Human studies have shown that chronically elevated adrenergic signaling leads to altered calcium homeostasis in the failing hypertrophied cardiac myocyte and results in an increase in intracellular calcium concentrations [28,29]. Studies based on human heart biopsies have shown that calcium-activated gene expression leads to an induction of a ‘fetal’ gene expression profile for the contractile proteins and directly contributes to contractile dysfunction and pathologic hypertrophy in the heart [28,29].

The fetal pattern of gene expression includes an upregulation of the fetal genes, β-myosin heavy chain and atrial natriuretic peptide (ANP), and a downregulation of the adult genes, α-myosin heavy chain and sarcoendoplasmic reticulum Ca^2+^-ATPase 2a (SERCA2a) [29,30]. ANP is secreted by the heart in response to volume expansion and leads to an increase in sodium excretion [31]. In agreement with this notion, we found increased concentrations of vanillactate, a metabolite that has previously been shown to be upregulated by sodium reduction in hypertensive subjects [32]. We also found elevated concentrations of dimethylglycine (DMG) in participants carrying pathogenic variants for HCM (Figure 1). Apoptosis of cardiomyocytes seems partly to depend on mitochondrial processes [33], and DMG metabolism inside the mitochondria may influence the production of nucleotides [34], potentially affecting the cell’s regenerative abilities.

We found that participants carrying the pathogenic HCM genetic variants showed predominantly decreased concentrations of long-chain polyunsaturated fatty acids (PUFAs) and lysophosphatidylcholines (LPC) containing PUFAs in their acyl chain (Figure 1). We also found decreased concentrations of the PUFAs eicosapentaenoate (EPA), arachidonate, and docosahexaenoate (DHA) in participants carrying pathogenic HCM genetic variants. In vitro studies have shown that EPA and DHA repress hypertrophic responses in cardiomyocytes by the direct inhibition of histone acetyltransferase activity, and that EPA attenuates endothelin 1 (ET-1)-induced cardiomyocyte hypertrophy [35,36].

We also found decreased concentrations of LPCs in the participants carrying pathogenic genetic variants for HCM (Figure 1). Hydrolysis of phosphatidylcholine by phospholipase A2 generates LPC and a fatty acid [37]. LPCs have potent cardiac effects, including inhibition of the release of atrial natriuretic peptide (ANP) [38]. Cardiac hypertrophy is a fundamental process of adaptation to an increased workload due to hemodynamic overload and is known to activate the cardiac ANP system, with a subsequent high plasma concentration as one of the cardiac compensatory mechanisms [31]. Ca^2+^, together with calcium-calmodulin kinase II, may be one of the most important factors affecting ANP secretion [39]. An increase in intracellular calcium, combined with an increased generation of reactive oxygen species (ROS), leads to the activation of Ca^2+^/calmodulin kinase II in the hypertrophied heart, triggering the structural deterioration of the myocardium and resulting in contractile dysfunction and arrhythmia in the failing heart [40].

**Figure 2:**
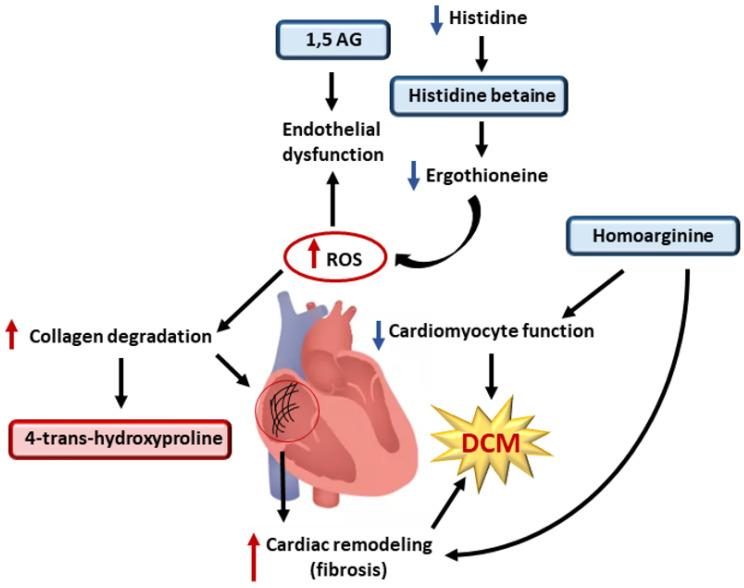
Metabolites involved in the generation of reactive oxygen species, collagen degradation, and cardiac remodeling in the carriers of pathogenic DCM variants. Red boxes indicate increased concentrations and blue boxes decreased concentrations of the metabolites in the carriers of the pathogenic genetic variants compared to the controls. Collagen degradation increases 4-trans-hydroxyproline concentrations and increases the rate of cardiac remodeling, leading to DCM. Decreased levels of histidine betaine lead to low concentrations of antioxidant ergothioneine, which increases the generation of ROS. ROS, in turn, increases collagen degradation and plays a role in endothelial dysfunction, which is also aggravated by decreased concentrations of 1,5 AG. Low concentrations of homoarginine decreases cardiomyocytes function and increases the rate of cardiac remodeling, triggering the development of DCM. Abbreviations: 1,5 AG, 1,5-anhydroglucitol; DCM, dilated cardiomyopathy; ROS, reactive oxygen species.

We found that the participants carrying pathogenic genetic variants for DCM had decreased concentrations of 1,5-anhydrogluticol (1,5-AG), a metabolite belonging to the carbohydrate pathway (Figure 2). Previous studies have shown that 1,5-AG concentrations are associated with vascular endothelial dysfunction [41] and that endothelial dysfunction is associated with the development of DCM [42]. We found that bilirubin concentration was decreased in carriers of the pathogenic DCM variants [43]. In agreement with our results, previous studies have shown that bilirubin concentrations are significantly decreased in patients with heart failure [44,45].

In our study, the carriers of a pathogenic DCM variant had decreased concentrations of metabolites belonging to the amino acid pathway, namely, homoarginine, histidine betaine, N-acetyltryptophan, and methylsuccinate (Figure 2). Decreased homoarginine concentration has been associated with dilatation and decreased function of the left ventricle in the general population [46]. Additionally, a previous study in mice has shown that decreased homoarginine concentrations may impair cardiomyocyte function [47]. Histidine betaine is a downstream metabolite of histidine generated by gut bacteria and a precursor of an antioxidant ergothioneine [48,49]. A previous study reported that plasma concentrations of histidine were reduced in patients with primary DCM, as compared to corresponding controls [43].

We found increased concentrations of trans-4-hydroxyproline in the carriers of pathogenic DCM variants. Trans-4-hydroxyproline is a major component of collagen [37], and trans-4-hydroxyproline is used as a parameter of collagen catabolism [37]. Increased ROS formation is also known to accelerate collagen degradation [37].

Overall, our findings indicate that the hypertrophy of cardiomyocytes in carriers of HCM variants is regulated not only by factors predisposing cardiac hypertrophy, but also by factors that attempt to counteract hypertrophy in response to hemodynamic loading (Figure 1). In the carriers of DCM variants, we found a metabolite profile compatible with vascular endothelial dysfunction, a decrease in antioxidant precursors, an increase in ROS generation, an increase in cardiac remodeling, and a high protein turnover in cardiomyocytes, indicating a disturbed collagen metabolism in carriers of DCM variants (Figure 2).

The strength of our study is a large size of our population-based study cohort and detailed analyses of genetic variants and 1098 metabolites. Additionally, we identified several novel metabolites associated with pathogenic genetic variants for HCM and DCM. A major limitation of our study is that both HCM and DCM are rare diseases, and therefore, the prevalence of these diseases is small, which makes it difficult to obtain statistically significant results. To improve the power of our study, we selected five controls for each case of HCM or DCM. Other limitations of our study are that only middle-aged and elderly Finnish men were included in the study. We do not know if the results are valid for women, all age groups, and other ethnic and racial groups. Therefore, our findings need to be replicated in other studies. Finally, our study is an association study that does not allow us to make causal conclusions from our results.

## 4. Materials and Methods

### 4.1. Subjects

The METSIM study is a randomly selected, population-based study comprising of 10,197 men recruited from Kuopio and surrounding communities in Eastern Finland, aged from 45 to 73 years at baseline [15]. The METSIM study was approved by the Ethics Committee of the University of Eastern Finland and Kuopio University Hospital and was conducted in accordance with the Declaration of Helsinki. Written informed consent was given by all participants.

Patients with HCM had either echocardiographic or Magnetic Resonance Imaging measurement of a left ventricular wall thickness. All of them had a left ventricular wall thickness at least 15 mm, and they did not have systemic hypertension. All patients with DCM had left ventricular or biventricular systolic dysfunction and dilatation that are not explained by abnormal loading conditions or coronary artery disease [4].

### 4.2. Whole Exome Sequencing and Classification of Genetic Variants for Cardiomyopathies

A total of 9928 participants of the METSIM study had whole exome sequencing results. The methods of sequencing have been previously described in detail [50]. We identified pathogenic and likely pathogenic genetic variants from known genes causing HCM or DCM and classified these variants according to the ACMG/AMP 2015 guidelines [51]. We validated the results by Sanger sequencing using a BigDye Terminator v1.1 Cycle Sequencing Kit and analysis using 3500XL Genetic Analyzer (Thermo Fisher Scientific Inc., Waltham, MA, USA). TaqMan Allelic Discrimination Assay (Applied Biosystems QuantStudio 5 Real-Time PCR System, Thermo Fisher Scientific Inc., Waltham, MA, USA) was performed to identify the Gln1061Ter variant of *MYBPC3*.

We included in our analyses only the variants located in the protein coding regions or in canonical splice sites.

### 4.3. Metabolomics

Non-targeted metabolomics profiling was performed at Metabolon, Inc. (Morrisville, NC, USA) on EDTA plasma samples obtained after an overnight fast from 8679 participants, as previously described in detail [51,52,53]. The Metabolon DiscoveryHD4 platform was applied to assay named metabolites. All samples were processed together for peak quantification and data scaling. We quantified raw mass spectrometry peaks for each metabolite using the area under the curve and evaluated overall process variability by the median relative standard deviation for endogenous metabolites present in all 20 technical replicates in each batch. We adjusted for variation caused by day-to-day instrument tuning differences and columns used for biochemical extraction by scaling the raw peak quantifications to the median for each metabolite by the Metabolon batch. We included 1098 metabolites in the statistical analysis.

### 4.4. Statistical Analyses

We conducted statistical analyses using SPSS (version 27, IBM Corp., Armonk, NY, USA). Metabolite levels were log-transformed and standardized to a mean of 0 and a standard deviation of 1. We also log-transformed all other variables having a skewed distribution for statistical analyses and present the data for continuous variables as mean ± standard deviation (SD). All metabolite concentrations were compared between the variant carriers and controls by one-way analysis of variance (ANOVA). *p* < 0.05 was considered statistically significant. Each carrier of a pathogenic or likely pathogenic genetic variant for HCM or DCM was matched with five controls (without a known HCM/DCM variant and disease phenotype) based on age and body mass index (BMI). The fuzz relaxation was 0.25 to the standard deviation (SD) of age (SD = 1.75) and BMI (SD = 1). The iteration was repeated until the desired number of controls were found (the case-to-control ratio 1:5).

## 5. Conclusions

In conclusion, we identified several novel metabolites associated with the pathogenic or likely pathogenic sarcomere genetic variants for the development of HCM and DCM.

## Figures and Tables

**Table 1:** Clinical and laboratory measurements in the participants with HCM and DCM and corresponding controls.

Variable	HCM (*n* = 36) *	Controls (*n* = 180)	*p*	DCM (*n* = 20)	Controls (*n* = 100)	*p*
Age, years	58.9 ± 7.2	58.9 ± 7.1	0.990	56.6 ± 7.5	56.9 ± 7.5	0.883
Body mass index, kg/m^2^	26.3 ± 3.7	26.2 ± 3.6	0.967	27.8 ± 3.8	27.7 ± 3.7	0.961
Systolic blood pressure, mmHg	143.4 ± 17.0	138.9 ± 16.9	0.137	132.3 ± 14.3	135.8 ± 15.8	0.376
Diastolic blood pressure, mmHg	88.5 ± 8.6	86.6± 9.8	0.230	86.4 ± 9.0	86.3 ± 8.9	0.954
Total triglycerides, mmol/L	1.22 ± 0.58	1.31 ± 0.73	0.480	1.41 ± 0.82	1.88 ± 3.92	0.852
Free fatty acids, mmol/L	0.36 ± 0.17	0.38 ± 0.15	0.377	0.36 ± 0.16	0.39 ± 0.22	0.523
hS-CRP (mg/L)	1.45 ± 1.47	2.32 ± 6.59	0.148	2.39 ± 1.68	2.20± 3.46	0.163

* Two participants did not have metabolomics data available.

**Table 2:** Pathogenic or likely pathogenic genetic variants for HCM and DCM in the METSIM study.

HCM Variant	HCM (*n* = 38)	DCM Variant	DCM (*n* = 20)
*MYH7*, Arg1053Gln	24	*TTN*, Trp24243Ter	1
*MYH7*, Arg941His	4	*TTN*, Leu24076Ter	1
*MYBPC3*, Gln1061Ter	3	*TTN*, Gln4151Ter	1
*MYBPC3*, c.655-2A > C	4	*TTN*, Gln25397Ter	1
*MYBPC3*, Gly853fs	1	*TTN*, Pro28547GlnfsTer12	2
*TPM1*, Asp175Asn	2	*TTN*, c.7057 + 2dup	4
		*TTN*, Ala35063CysfsTer6	1
		*TTN*, Trp29474Ter	1
		*TTN*, Arg22499Ter	4
		*TTN*, c.67348 + 1G > A	1
		*TTN*, Arg27509Ter	1
		*TTN*, Gln23834Ter	2

**Table 3:** Statistically significant differences in metabolite concentrations between the carriers (Cases) and non-carriers (Controls) of the pathogenic or likely pathogenic HCM variants.

HMDB	Subclass	Metabolite	Cases			Controls				
Amino Acids			*n*	Mean	SD	*n*	Mean	SD	*p*	Novel
-	ɣ-glutamyl amino acid	Gamma-glutamyl-alpha-lysine	36	−0.055	0.096	179	0.017	0.113	4.9 × 10^−4^	Yes
HMDB0000913	Tyrosine Metabolism	Vanillactate	28	0.123	0.250	158	−0.003	0.218	0.006	Yes
HMDB0004246	Oligopeptide	Des-arg 9-bradykinin	5	0.430	0.674	36	−0.053	0.303	0.008	Yes
HMDB0000092	Glycine, Serine and Threonine Metabolism	Dimethylglycine	36	0.049	0.136	180	−0.005	0.109	0.009	Yes
**Lipids**										
**Fatty acids**										
HMDB0000321	Medium-chain FA	2-hydroxyadipate	14	0.141	0.323	46	−0.034	0.162	0.008	Yes
HMDB0031057	Long-chain FA	2-hydroxypalmitate	36	−0.033	0.095	180	0.013	0.086	0.005	Yes
HMDB0061859	Long-chain FA	(14 or 15)-methylpalmitate (a17:0 or i17:0)	36	−0.060	0.192	176	0.019	0.147	0.006	Yes
HMDB0001999	Long-chain PUFA	Eicosapentaenoate (EPA; 20:5n3)	36	−0.104	0.227	180	0.004	0.228	0.010	Yes
HMDB0001043	Long-chain PUFA	Arachidonate (20:4n6)	36	−0.077	0.140	180	0.001	0.130	0.001	Yes
HMDB0002183	Long-chain PUFA	Docosahexaenoate (DHA; 22:6n3)	36	−0.104	0.227	180	0.002	0.208	0.006	Yes
**Glycerophospholipids**										
-	Phosphatidylcholine (PC)	1-stearoyl-2-docosahexaenoyl-GPC (18:0/22:6)	36	−0.090	0.162	180	−0.009	0.163	0.007	Yes
-	Lysophosphatidylcholine	2-docosahexaenoyl-GPC (22:6)	34	−0.107	0.183	164	0.014	0.172	2.8 × 10^−4^	Yes
HMDB0010384	Lysophosphatidylcholine	1-stearoyl-GPC (18:0)	36	−0.048	0.090	180	0.007	0.092	0.001	Yes
HMDB0010403	Lysophosphatidylcholine	1-docosapentaenoyl-GPC (22:5n6) *	34	−0.117	0.232	158	0.021	0.221	0.001	Yes
N/A	Lysophosphatidylcholine	1-nonadecenoyl-GPC (19:1)	35	−0.066	0.156	168	0.027	0.162	0.002	Yes
HMDB0010404	Lysophosphatidylcholine	1-docosahexaenoyl-GPC (22:6)	36	−0.088	0.197	180	0.009	0.178	0.004	Yes
HMDB0010390	Lysophosphatidylcholine	1-arachidoyl-GPC (20:0)	36	−0.051	0.119	178	0.013	0.129	0.007	Yes
HMDB0061699	Lysophosphatidylcholine	2-arachidonoyl-GPC (20:4)	34	−0.078	0.168	173	0.005	0.143	0.009	Yes
N/A	Choline-lysoplasmalogen	1-palmityl-GPC (O-16:0)	36	−0.060	0.136	180	0.025	0.142	0.001	Yes
HMDB0011149	Choline-lysoplasmalogen	1-stearyl-GPC (O-18:0)	36	−0.052	0.128	175	0.015	0.128	0.004	Yes
HMDB0010407	Choline-lysoplasmalogen	1-(1-enyl-palmitoyl)-GPC (P-16:0) *	36	−0.048	0.129	180	0.016	0.126	0.006	Yes
HMDB0061690	Lysophosphatidylinositol	1-arachidonoyl-GPI (20:4)	36	−0.076	0.135	180	−0.004	0.123	0.002	Yes
**Other metabolites**										
HMDB0029968	Food Component/Plant	Ethyl beta-glucopyranoside	35	0.194	0.443	178	−0.035	0.421	0.004	Yes

**Table 4:** Statistically significant differences in metabolite concentrations between the carriers (Cases) and non-carriers (Controls) of the pathogenic or likely pathogenic DCM variants.

HMDB	Subclass	Metabolite	Cases			Controls				
Carbohydrate			*n*	Mean	SD	*n*	Mean	SD	*p*	Novel
HMDB0002712	Glycolysis, Gluconeogenesis, and Pyruvate Metabolism	1,5-anhydroglucitol	20	−0.137	0.300	100	−0.008	0.107	8.9 × 10^−4^	Yes
**Lipids**										
HMDB0000054	Bilirubins	Bilirubin (Z,Z)	20	−0.060	0.162	100	0.049	0.178	0.012	Yes
**Amino acids**										
HMDB0000670	Urea cycle; Arginine and Proline Metabolism	Homoarginine	20	−0.076	0.130	100	0.031	0.165	0.007	No
HMDB0029422	Histidine pathway	Histidine betaine (hercynine)	20	−0.189	0.328	85	0.057	0.373	0.008	Yes
HMDB0000725	Urea cycle; Arginine and Proline Metabolism	Trans-4-hydroxyproline	20	0.097	0.111	100	0.000	0.163	0.012	Yes
HMDB0013713	Tryptophan Metabolism	N-acetyltryptophan	20	−0.076	0.133	99	0.003	0.128	0.014	Yes
HMDB0001844	Leucine, Isoleucine and Valine Metabolism	Methylsuccinate	19	−0.082	0.180	95	0.000	0.128	0.019	Yes

## Data Availability

The data presented in this study are available on request from the corresponding author. The data are not publicly available due to preserving the confidentiality of the participants.
